# Inflammatory Breast Cancer: The Secretome of HCMV^+^ Tumor-Associated Macrophages Enhances Proliferation, Invasion, Colony Formation, and Expression of Cancer Stem Cell Markers

**DOI:** 10.3389/fonc.2022.899622

**Published:** 2022-06-30

**Authors:** Hossam Taha Mohamed, Aya Ali El-Sharkawy, Mohamed El-Shinawi, Robert J. Schneider, Mona Mostafa Mohamed

**Affiliations:** ^1^ Zoology Department, Faculty of Science, Cairo University, Giza, Egypt; ^2^ Faculty of Biotechnology, October University for Modern Sciences and Arts, Giza, Egypt; ^3^ Department of General Surgery, Faculty of Medicine, Ain Shams University, Cairo, Egypt; ^4^ Galala University, Suez, Egypt; ^5^ Department of Microbiology, School of Medicine, New York University, New York, NY, United States; ^6^ Sector of International Cooperation, Galala University, Suez, Egypt

**Keywords:** inflammatory breast cancer, breast cancer stem cell, human cytomegalovirus, tumor-associated macrophages, secretome

## Abstract

Inflammatory breast cancer (IBC) is a highly aggressive phenotype of breast cancer that is characterized by a high incidence early metastasis. We previously reported a significant association of human cytomegalovirus (HCMV) DNA in the carcinoma tissues of IBC patients but not in the adjacent normal tissues. HCMV-infected macrophages serve as “mobile vectors” for spreading and disseminating virus to different organs, and IBC cancer tissues are highly infiltrated by tumor-associated macrophages (TAMs) that enhance IBC progression and promote breast cancer stem cell (BCSC)-like properties. Therefore, there is a need to understand the role of HCMV-infected TAMs in IBC progression. The present study aimed to test the effect of the secretome (cytokines and secreted factors) of TAMs derived from HCMV^+^ monocytes isolated from IBC specimens on the proliferation, invasion, and BCSC abundance when tested on the IBC cell line SUM149. HCMV^+^ monocytes were isolated from IBC patients during modified radical mastectomy surgery and tested *in vitro* for polarization into TAMs using the secretome of SUM149 cells. MTT, clonogenic, invasion, real-time PCR arrays, PathScan Intracellular Signaling array, and cytokine arrays were used to characterize the secretome of HCMV^+^ TAMs for their effect on the progression of SUM149 cells. The results showed that the secretome of HCMV^+^ TAMs expressed high levels of IL-6, IL-8, and MCP-1 cytokines compared to HCMV^-^ TAMs. In addition, the secretome of HCMV^+^ TAMs induced the proliferation, invasion, colony formation, and expression of BCSC-related genes in SUM149 cells compared to mock untreated cells. In addition, the secretome of HCMV^+^ TAMs activated the phosphorylation of intracellular signaling molecules p-STAT3, p-AMPKα, p-PRAS40, and p-SAPK/JNK in SUM149 cells. In conclusion, this study shows that the secretome of HCMV^+^ TAMs enhances the proliferation, invasion, colony formation, and BCSC properties by activating the phosphorylation of p-STAT3, p-AMPKα, p-PRAS40, and p-SAPK/JNK intracellular signaling molecules in IBC cells.

## Introduction

IBC is a highly metastatic and lethal subtype of breast cancer presenting with high incidence among young women in the Middle East and North Africa (1). TAMs of IBC patients were found to contribute to high levels of growth factors and cytokines in the TME and enhance metastasis *via* different mechanisms (2, 3).

In breast cancer, studies showed that the incidence of BCSCs plays a key role in recurrence and metastasis, which is associated to a certain extent with the presence of macrophages in the TME (4). For instance, β-Catenin activation attracts monocytes and induces the expression of CCL2 which changes monocytes to macrophages (needs a reference). BCSCs are associated *in-vivo* with increased metastasis activity promoted by β-Catenin-mediated CCL2 and TAMs (CD163) (5). TAMs in breast cancer TME induce stem cell properties *via* different mechanisms (6). Our previous studies identified different molecular, cellular, viral, immunological, and stem cell drivers involved in the poor prognosis of IBC (3, 7–10). For instance, we have been studying the role of TAMs in IBC progression. We showed that monocytes/macrophages identified as CD14^+^ cells highly infiltrate IBC cancer tissues and are localized with high density around IBC tumor emboli, clusters of highly metastatic IBC cells (3). The detected cytokines secreted by TAMs suggest a role for viral infection in the etiology of IBC (3).

Even though several studies showed links between viral infection and poor breast cancer prognosis (11), a causative role for viral infection in breast cancer development and progression is still uncertain, including in IBC. Previous studies by the authors assessed the incidence of different viral DNAs including HPV-16 and -18, HCMV, HBV, HSV-1, HSV-2, and HHV-8 in the cancer tissues of IBC versus non-IBC patients (9). Results showed that the incidence of multiple viral DNAs in IBC tissues was higher compared to non-IBC tissues, with the most prevalent detected viruses identified as HCMV and HPV-16. Moreover, detection of the DNA of different viruses positively correlated with the upregulation of the Ki-67 proliferation marker in IBC cells. Our results suggested a significant association between viral infection and IBC disease etiology (9). Herpes virus association with IBC may not be unique (7). HCMV is detected in more aggressive forms of malignancies including colorectal cancer (12), malignant glioblastoma (13), and breast cancer (14, 15). Regarding IBC, we found that HCMV-DNA is more prevalent in the cancer tissues of IBC but not in the adjacent non-cancer tissues (8). HCMV-infected IBC cancer tissues also exhibit higher expression and activation (phosphorylation) of NF-κB/p65 in cancer tissues of IBC patients than those of non-IBC patients (8), although a causal relationship to increased HCMV presence has not been established. In addition, we showed that certain polymorphisms in HCMV genotypes may be associated with increased involvement of HCMV in IBC disease etiology (9).

Monocytes/macrophages are a major site of persistence of HCMV in the peripheral blood (16). Whereas HCMV does not replicate in monocytes, primary HCMV infection to monocytes induces differentiation and biological turnover of monocytes to tissue macrophages. In addition, infected macrophages serve as “mobile vectors” for virus spreading and dissemination to different organs mainly by trans-endothelial migration (17). CD14^+^ monocytes were found to be a reservoir for HCMV latency (18). Furthermore, monocyte HCMV infection induces the differentiation of monocytes into macrophages characterized by the expression of Toll-like receptor (TLR) and inflammatory cytokines TNF-α, IL-6, and IL-8 (19). Notably, and possibly related, gene expression profile studies showed that TLR and IL-6 are more highly expressed in IBC patients compared to non-IBC patients (20, 21). Among subpopulations of macrophages associated with viral infection are macrophages expressing the marker MAC387 (22). MAC387 is associated with poor prognosis and increased distant metastasis of cholangiocancers (CCA) (23). HCMV^+^ macrophages are associated with HCMV viral replication and are characterized by significantly high expression of TNF-α, IL-6, and. IL-8 at gene and protein levels in response to stimulation by TLR4 ligand (LPS) compared with mock uninfected macrophages (19). Using *in-vitro* co-culture models seeding MRC-5 cells infected with HCMV-encoded UL128 protein (HCMV-UL128^+^) with peripheral blood mononuclear cells (PBMCs), significantly induced secretion of IL-6 and TNF-α in the culture media was found compared to those co-cultured with HCMV-UL128^-^ (24). IL-6 binds to tumor cell transmembrane IL-6 receptors (IL-6R) and subsequently dimerizes with glycoprotein 130 (gp130), a transmembrane protein that behaves like a signal transducer following cytokine engagement. This binding activates the Janus Kinases/Signal Transducers and Activators of Transcription (JAK/STAT) pathway, which ultimately induces invasion and motility of cancer cells (25). This links back to BCSCs, because the Jak-STAT pathway modulates BCSC activity (26).

Several *in vitro* studies on different cancer cell lines revealed that HCMV infection in the TME promotes stemness and an epithelial-to-mesenchymal transition (EMT), leading to increased cancer aggressiveness (19). Moreover, HCMV infection in breast cancer cells inhibits EMT and promotes the mesenchymal-epithelial transition (MET), which is necessary for colonization of metastases (27). Supporting this hypothesis, it was found that 96% of brain metastases derived from primary breast cancers were HCMV^+^, while adjacent normal tissues are virus-free (28). Moreover, high expression of GM-CSF is associated with the prevalence of CCL18-secreting macrophages and the realization of EMT by breast cancer cells (29).

In the present study, we showed that HCMV^+^ IBC tissues are characterized by higher infiltration of CD163^+^ and MAC387^+^ TAMs compared to HCMV^-^ IBC tissues. SUM149 cells stimulated by the secretome of HCMV^+^ TAMs showed an increase in proliferation, invasion, colony formation, and expression of BCSC-related genes compared to mock infected cells. A probable mechanism of action involves activation of p-STAT3, p-AMPKα, p-PRAS40, and p-SAPK/JNK intracellular signaling molecules. These results highlight a potential critical onco-modulatory role of HCMV infection in IBC.

## Materials and Methods

### Patient Samples

The study protocol was approved by the Institutional Review Board (IRB#00006379), Faculty of Medicine, Ain Shams University, Egypt. A total of 28 IBC patients were enrolled in the present study. IBC patients were clinically and pathologically diagnosed as previously described (3, 30). Patients suffering from Hepatitis or AIDS or COVID-19 or auto-immune diseases were excluded from this study. Before participation, every patient signed a consent form, including approval for publication of the study results.

### Serological Assay

Five ml of peripheral blood was collected from each IBC patient in red-top tubes, which contained no anticoagulant or preservative (Greiner Bio-one, Kremsmünster, Austria) for serological diagnosis of HCMV IgG antibodies. Blood was collected and centrifuged at 1500 rpm for 10 min to isolate serum for serological tests. IgG antibodies against HCMV were measured in serum samples using HCMV IgG Chemiluminescence detection kit (Diasorin, Liaison, Italy) and a Liaison chemiluminescence device. The kit measures HCMV IgG antibody concentrations which are expressed as IU/ml. Samples with HCMV IgG concentrations equal to or more than 0.6 IU/ml were considered positive (31).

### Isolation of TME CD14^+^ Monocytes

During axillary dissection in modified radical mastectomy (MRM) operations, 10 ml of blood was withdrawn from the identified axillary tributaries in a heparinized syringe with an angular needle as described before (32). Plasma was collected from the whole blood sample *via* centrifugation at 1500 rpm for 20 min. Mononuclear cells were separated from the precipitated blood content by Ficoll-Hypaque density gradient centrifugation (Lonza, ME, USA) at 1500 rpm for 30 min. The buffy coat layer containing mononuclear cells was separated and washed twice with PBS. The TME CD14^+^ monocytes were purified from the mononuclear cells using “EasySep™ Human Monocyte Enrichment Kit without CD16 Depletion” (StemCell Technologies, Vancouver, Canada). Purified TME CD14^+^ monocytes were grown overnight in Petri dishes (35 X 10 mm) at a concentration of (1 x 10^6^ cells/ml) in RPMI media with 1% of penicillin/streptomycin antibiotic mixture in 3% fetal bovine serum (FBS) and incubated at 37°C in 5% CO_2_ for 24 h. Media conditioned by TME CD14^+^ TAMs secretions were collected as TAMs secretome and concentrated 1:100 using Vivaspin™ protein concentrator column (Sartorius, Goettingen, Germany) with 10,000 molecular weight cutoff value (MWCO value), and its protein content was determined *via* Bradford assay (Biorad Laboratories, CA, USA) and measured by Multiskan SkyHigh microplate spectrophotometer (ThermoFisher Scientific, MA, USA). Cells were aliquoted and stored at −80°C for DNA, RNA, and protein extraction.

### Nested PCR

DNA was extracted from purified IBC cancer tissues and isolated TME CD14+ monocytes/macrophages (1 x 106 cells/ml) of HCMV seropositive and seronegative IBC patients using GeneJETTM Genomic DNA purification Kit (Thermo Scientific, MA, USA). The extracted DNA was used in nested PCR for detecting the fourth exon of the HCMV Immediate Early (IE) gene was performed as described before (8, 33). Amplified PCR products (293 bp) were visualized on 1.5% agarose gels (Bio Basic INC, ON, Canada) stained with ethidium bromide, and photographed by the GBOX-F3 gel documentation system (Syngene, MD, USA).

### Immunohistochemistry

Immunohistochemical (IHC) staining was performed after chemical dewaxing of 4 μm thick formalin-fixed paraffin-embedded (FFPE) tissue sections as described before (30, 34) using antibodies for CD14 (diluted at 1:50 in PSA) (Chemicon, CA, USA), CD68 (diluted at 1:50 in PSA) (M0814) from Dako (Agilent, CA, USA), CD163 (1:500) (Abcam, Cambridge, UK), and MAC387 (1µg/ml) (Abcam, Cambridge, UK). IHC Staining was carried out by adding 100 µl of DAB+ chromogen diluted at 1:50 in substrate buffer [(EnVision+ Dual Link System-HRP (DAB+)] for 10 min. Finally, tissue specimens were washed in phosphate buffer saline (PBS), the nuclei were counterstained with hematoxylin and mounted using Permount^®^ (Fisher Scientific, PA, USA) for microscopic examination. Negative control slides were run in parallel with each marker where primary antibody was replaced by PBS. The stained area fractions were calculated using ImageJ software (National Institutes of Health, Bethesda, MD, USA) (7, 8).

### Microscopic Examination of Polarized HCMV^+^ TAMs

For cellular morphology of *in vitro* polarized HCMV^+^ TAMs, microscopic images were taken at 40x magnification. Three distinct morphologic subtypes were identified, (M0) were small/roundish cells characterized by a cellular diameter ≤ 10 μm with the absence of cytoplasmic projections on the cell surface, (M1) were enlarged amoeboid cells characterized by a cellular diameter ≥ 10 μm and presence of many delicate cytoplasmic extensions on the cell surface with visible intracellular vacuoles, and (M2) demonstrated a large and elongated “spindeloid” cells with a cellular diameter between 10 to 30 μm and cytoplasmic extensions on the apical ends of the cell bodies (35, 36).

### Gene Expression Signature of *In Vitro* Polarized HCMV^+^ TAMs

Studies confirmed the effect of the secretome of SUM149 on the polarization status of *in vitro* polarized HCMV^-^ and HCMV^+^ TAMs. Based on previous studies (37–44), we carefully designed and customized a PCR array that included 43 macrophages polarization-related genes (Vivantis, Selangor, Malaysia) to analyze the gene expression signature of *in vitro* polarized HCMV^-^ and HCMV^+^ TAMs. Total RNA was purified from mock and polarized HCMV^-^ and HCMV^+^ TAMs using QIAzol lysis reagent (Qiagen, Hilden, Germany). The total RNA concentration was measured by Multiskan SkyHigh microplate spectrophotometer (ThermoFisher Scientific, MA, USA), and RNA integrity was tested by separating the RNA on a 1% standard agarose gel and examining the ribosomal RNA bands. One µg of RNA was transcribed into complementary DNA (cDNA) using a High-Capacity cDNA Reverse Transcription Kit (ThermoFisher Scientific, MA, USA). A list of all primers included in this array is described in **Supplementary Table S1**. The quantitative real-time PCR was conducted using SYBR Green dye (Qiagen, Hilden, Germany) and gene expression levels were measured using AriaMX System (Agilent, CA, USA).). PCR thermal profile was 95°C initial denaturation for 10 minutes, followed by 40 cycles at 94°C for 15 seconds and 60°C for 1 minute. The obtained CTs were normalized to the CTs of *18S*, *ACTB*, *B2M, GAPDH*, and *HPRT* housekeeping genes and analyzed using Pfaffl’s method (45).

### Human Cytokine Antibody Array

Proteomic composition of the secretome of HCMV^-^ and HCMV^+^ TAMs were characterized quantitatively using RayBio™ human cytokine antibody array 3 (RayBiotech Life, GA, USA). Briefly, antibody array membranes were incubated in blocking buffer for 1 h and then incubated overnight at 4°C with 1 mL of the secretome of HCMV^-^ and HCMV^+^ TAMs (2000 µg/ml). After washing, membranes were incubated with the biotinylated antibody cocktail for 2 h at room temperature with shaking, and then incubated with HRP-streptavidin at room temperature for 2 h. Finally, membranes were developed using the chemiluminescence detection reagent provided with the kit. The quantification of each cytokine was achieved by densitometry analysis using ImageJ software (National Institutes of Health, Bethesda, MD, USA) as we described before (46).

### Cell Line

The SUM149 breast cancer cell line was provided by Prof. Schneider’s lab in the Department of Microbiology, New York University School of Medicine, USA, to be used in this study representing the IBC. SUM149 cells were cultured in HAM’s F12 medium supplemented with 5% fetal bovine serum (FBS), 5mM HEPES, 1µg/ml Hydrocortisone, 5µg/ml insulin, and 1% of penicillin/streptomycin antibiotic mixture. SUM149 cells were incubated in a humidified atmosphere at 37°C in 5% CO_2_. SUM149 cells were authenticated by STR profiling and routinely checked for mycoplasma contamination.

### Culturing of SUM149 cells in culture media with/without the secretome of HCMV+ TAMs

SUM149 cells were cultured in 6 well plates (3x10^5^ cell/well) in 2 ml Ham/s F12 culture medium supplemented with 5% FBS and incubated for 48h in a humidified atmosphere at 37°C in 5% CO_2_. When cells reached 75-80% confluency, they were seeded in culture media containing 1% FBS and conditioned with concentrated secretome of HCMV^-^ and HCMV^+^ TAMs (2000 ng/mL). Control SUM149 cells (Mock) were seeded in a complete culture media with 1% FBS. Cells were incubated for 72 h in a 5% CO_2_ humidified incubator at 37 °C and then culture media was discarded. Cells were washed twice with room temperature PBS and aliquoted to be stored at −80°C for RNA and protein extraction.

### Cell Proliferation Assay

MTT assay was used to test the effect of the secretome of HCMV^-^ and HCMV^+^ TAMs on the proliferation of SUM149 cells. Mock and stimulated SUM-149 cells were placed in a 96-well plate at a density of 4×10^3^/well and were incubated for 48 hours under optimum conditions. After that, 20 μL (5 g/L) of MTT (3-(4,5-dimethyl-2-thiazolyl)- 2,5-diphenyl-2-H-tetrazolium bromide) reagent was added to the designated wells. After a 4 h incubation, the MTT formazan precipitate was dissolved in dimethylsulphoxide (DMSO) (150 μL/well, Sigma-Aldrich, St. Louis, MO, USA) in a shaker for 5 min before reading the absorbance at 570 nm using a 96-well plate reader (Bio-Rad, Winooski, VT USA) (47–49).

### Transwell Cell Invasion Assay

To test the effect of the secretome of HCMV^-^ and HCMV^+^ TAMs (2000 ng/mL) on the invasive properties of SUM149 cells, we used Corning^®^ BioCoat™ Matrigel^®^ Invasion Chambers (Corning, MA, USA). According to the kit guidelines, cells that invade should be able to secrete proteases and degrade the Matrigel matrix-coat insert of the chamber. Thus, the invasion assay was conducted as described in the instruction guidelines of the kit. SUM149 cells were grown in the upper chamber with Ham’s F12 culture media 1% FBS in the lower chamber (control). The secretome of HCMV^-^ and HCMV^+^ TAMs (2000 ng/ml) were added to the lower chamber. Invasion chambers were incubated in humidified CO_2_ incubator at 37°C for 24 h. The percentage of invasion was calculated by counting the number of invaded cells in response to the chemotactic factor and dividing them by the control and multiplying by 100 as described by the equation in the kit manual.

### Clonogenic Assay

To test the effect of the TAM secretions prepared from HCMV^-^ and HCMV^+^ on the clonogenic ability of SUM149 cells, 1000 mock and stimulated SUM-149 cells were seeded in six-well plates and maintained in Ham-F12 with 5% FBS for 10–14 days as previously performed (50). Cells were washed with PBS, fixed in methanol for 20 min, and stained with 0.05% crystal violet for 20 min and the excess stain was removed by water. The stained colonies were counted as described before (10, 51).

### RT^2^ Profiler PCR Arrays for Detection of Extracellular Matrix and Cell Adhesion Molecules Expressed Genes

RT^2^ extracellular matrix and adhesion molecule PCR array (Qiagen, Hilden, Germany) was used to study the gene expression profiles of 84 extracellular matrix and cell adhesion molecules in SUM149 cells seeded in growth media conditioned by the secretome of HCMV^-^ and HCMV^+^ TAMs. Briefly, PCR array was performed using AriaMX System (Agilent, CA, USA) in a 25 µL total volume using RT^2^ SYBR Green Master Mix (Qiagen, Hilden, Germany). PCR thermal profile was 95°C initial denaturation for 10 minutes, followed by 40 cycles at 94°C for 15 seconds and 60°C for 1 minute. Data were analyzed using the Qiagen Gene globe web tool (https://geneglobe.qiagen.com/analyze/) after normalization to *ACTB*, *B2M, GAPDH*, *HPRT1*, and *RPLP0* housekeeping genes.

### Breast Cancer Stem Cell-Related Genes PCR Array

Based on previous studies (52–56), we carefully designed and customized BCSC-related gene PCR array including 43 genes (Vivantis, Selangor, Malaysia) to test the effect of the secretome of HCMV^-^ and HCMV^+^ TAMs on the expression of BCSC-related genes in SUM149 cells. The quantitative real-time PCR was conducted using SYBR Green dye (Qiagen, Hilden, Germany) and gene expression levels were measured using AriaMX System (Agilent, CA, USA). A list of all primers included in this array is described in **Supplementary Table S2**. PCR thermal profile was 95°C initial denaturation for 10 minutes, followed by 40 cycles at 94°C for 15 seconds and 60°C for 1 minute. The obtained CTs were normalized to the CTs of *18S*, *ACTB*, *B2M, GAPDH*, and *HPRT* housekeeping genes and analyzed using Pfaffl’s method (45).

### Enrichment Analysis of Differentially Expressed Genes

GeneMANIA (http://genemania.org/) is an online tool that provides large information about protein-DNA, protein-protein, and genetic interactions, reactions, pathways, and protein domains of related genes. Herein, we used GeneMANIA to compare the correlation between DEGs in SUM149 cells stimulated by the secretome of HCMV^+^ TAMs and neighboring related genes. In addition, Metascape (http://metascape.org/gp/index.html#/main/step1) is a public online analysis database that was used for functional annotation and enrichment analysis based on Gene Ontology (GO), Kyoto Encyclopedia of Genes and Genomes (KEGG), Reactome, canonical pathways, and hallmark gene terms for differentially expressed genes (DEGs) in SUM149 cells stimulated by the secretome of HCMV^+^ TAMs. The analysis was based on the relevant parameters as follows: minimum overlap, 3; P-value cutoff, 0.01 and minimum enrichment 1.5. All protein-protein interactions among input genes were extracted from the PPI data source and formed a PPI network. GO enrichment analysis was applied to the network to extract “biological meanings”. The MCODE algorithm was then applied to this network to identify neighborhoods where proteins are densely connected (57–60).

### Intracellular Signaling Array

PathScan^®^ Intracellular Signaling Array Kit (Cell signaling technology, MA, USA) was used to study the effect of the secretome of HCMV^-^ and HCMV^+^ TAMs on activating different signaling molecules in SUM149 cells. Mock and stimulated SUM149 cells’ lysates were diluted to 0.2 – 1.0 mg/mL using array diluent buffer before performing the Pathscan array. Pathscan glass slides were incubated in blocking buffer for 1h at room temperature with shaking. After washing, 50 - 75 μL of each cell lysate was added to each well and covered with sealing tape and incubated overnight at 4°C on an orbital shaker. After that, cell lysate was removed and the Pathscan glass slides were washed 3 successive times using the washing buffer provided with the kit. Detection Antibody Cocktail was added to each well and covered with sealing tape and incubated for 1 h at room temperature with shaking. After washing 3 times, 75 μL (1X) HRP-linked Streptavidin was added to each well and covered with sealing tape, and incubated for 30 minutes at room temperature with shaking. Subsequently, Pathscan glass slides were washed 3 successive times using washing buffer and covered with LumiGLO^®^/Peroxide reagent. Finally, Pathscan slides were transferred to the chemiluminescent development folder and images taken using ChemiDoc XRS+ Gel Imaging System (Bio-Rad, CA, USA). Relative density values were calculated *via* densitometric methods using ImageJ software (National Institutes of Health, MD, USA).

### Statistical Analysis

The Statistical Package of the Social Sciences software (SPSS, Chicago, IL, USA), version 22.0 was used for data analysis. The data were presented as the mean ± standard deviation (SD). In addition, differences among two groups of variables were evaluated using Student’s t-test and Chi-square test. The statistical difference between more than two groups was evaluated using one-way ANOVA followed by Tukey’s HSD *Post Hoc* tests. The level of significance was set at P < 0.05 (61–63).

## Results

### HCMV-DNA Detection in TME Derived Monocytes of HCMV^+^ IBC Patients

**Figure 1:**
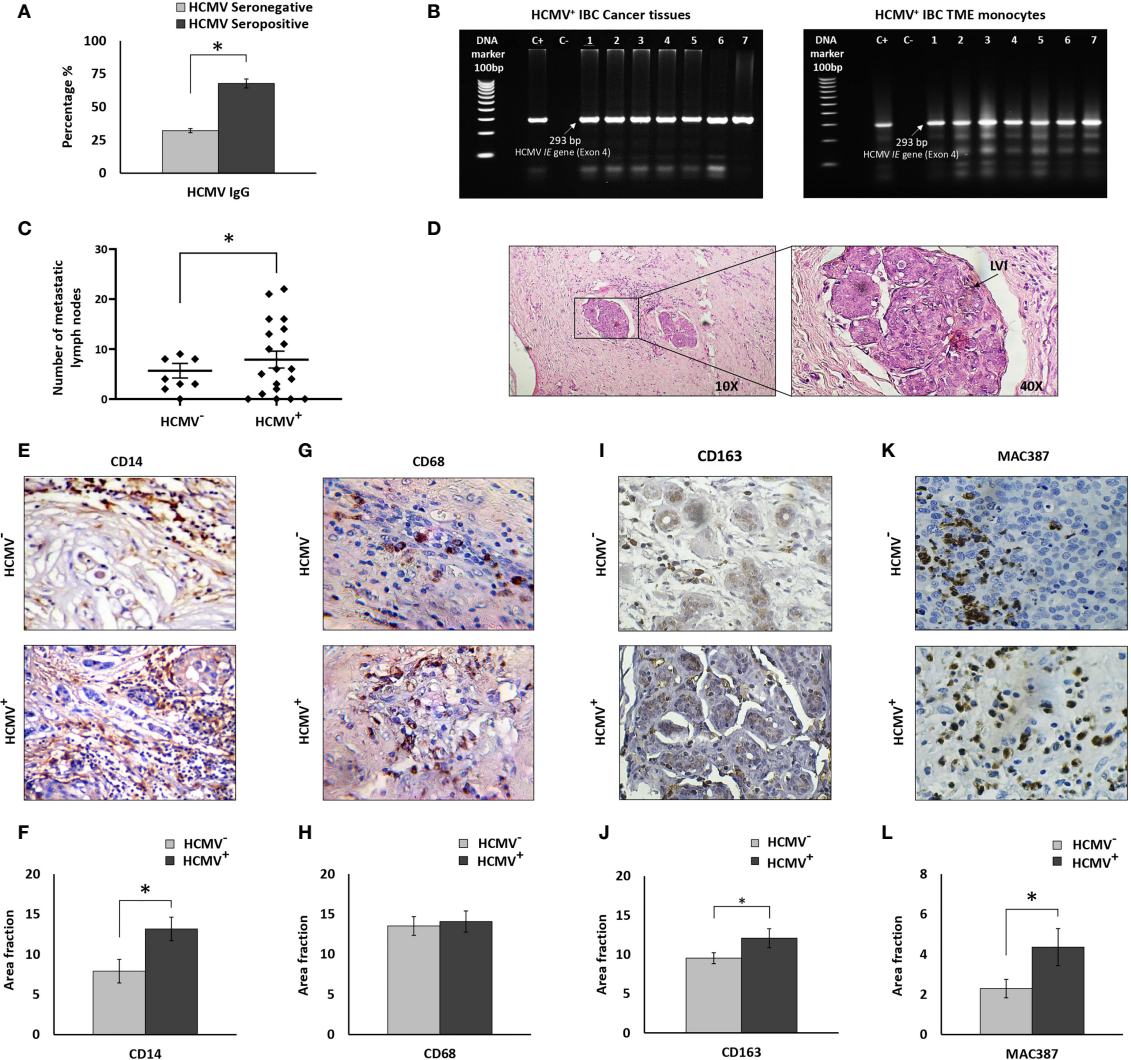
Prevalence of HCMV-DNA among IBC patients and IHC staining of IBC cancer tissues against CD14, CD68, CD163, and MAC387. **(A)** Bars represent prevalence of HCMV IgG among IBC patients. **(B)** Representative agarose gel electrophoresis showing amplicons of HCMV Immediate Early (*IE*) gene nested PCR (296 bp) among HCMV^+^ IBC cancer tissues and isolated TME CD14^+^ monocytes. **(C)** Scatter plot showing the number of metastatic lymph nodes among HCMV^-^ versus HCMV^+^ IBC patients. **(D)** Microscopic images of H and E stained paraffin embedded tissue section of HCMV^+^ IBC patients showing invasion of cancer cells to lymphatic vessels. **(E, F)** Microscopic images of CD14 stained paraffin-embedded tissue sections in HCMV^-^ versus HCMV^+^ IBC cancer tissues and bars showing a significantly high prevalence of CD14^+^ monocytes among HCMV^+^ compared to HCMV^-^ IBC cancer tissues. **(G, H)** Microscopic images of CD14 stained paraffin-embedded tissue sections in HCMV^-^ versus HCMV^+^ IBC cancer tissues. Bars show no significant difference in the prevalence of CD68^+^ TAMs among HCMV^+^ compared to HCMV^-^ IBC cancer tissues. **(I, J)** Microscopic images of CD163 stained paraffin-embedded tissue sections in HCMV^-^ versus HCMV^+^ IBC cancer tissues. Bars show a significantly high prevalence of CD163^+^ TAMs among HCMV^+^ compared to HCMV^-^ IBC cancer tissues. **(K, L)** Microscopic images of MAC387 stained paraffin-embedded tissue sections in HCMV^-^ versus HCMV^+^ IBC cancer tissues and bars showed a significantly high prevalence of MAC387^+^ TAMs among HCMV^+^ compared to HCMV^-^ IBC cancer tissues. Data represented the mean of ± SD. *P* values were calculated using Student *t*-test, where * represented (*P* < 0.05).

Results of HCMV IgG serological assay of peripheral blood showed that 19 out of 28 IBC patients were HCMV IgG positive (**Figure 1A**). HCMV-DNA of the fourth exon of the HCMV *IE* gene was detected in all cancer tissues of HCMV seropositive IBC patients (**Figure 1B**). Moreover, HCMV-DNA was detected in the isolated TME monocytes of HCMV seropositive IBC patients (**Figure 1B**). In contrast, HCMV-DNA was not detected in the cancer tissues or the isolated TME derived monocytes of all HCMV seronegative IBC patients. However, it should be noted that we did not detect HCMV-DNA in 4 out of 19 (21%) TME derived monocytes isolated from HCMV seropositive IBC patients.

### Clinical and Pathological Characterization of HCMV^-^ and HCMV^+^ IBC Patients

Clinical and pathological characterizations of HCMV^-^ and HCMV^+^ IBC patients are described in **Table 1**. Statistical analysis revealed that there are no significant differences in age, tumor size, tumor grade, status of lymph node metastasis, hormonal receptors, and receiving neoadjuvant chemotherapy between HCMV^-^ and HCMV^+^ IBC patients. On the contrary, the number of metastatic lymph nodes and the presence of lymphovascular invasion in HCMV^+^ IBC patients were statistically significantly higher (*P*= 0.03 and 0.021, respectively) compared to HCMV^-^ IBC patients (**Figures 1C, D**). This confirmed our previous results (8).

**Table 1:** Clinical and pathological characterization of HCMV^-^ versus HCMV^+^ IBC patients.

Characteristic	HCMV^-^(N = 9)	HCMV^+^(N = 19)	*P* value
**Age [year]**			
Range Mean ± SD	33-75 44.9 ± 12.4	33-70 49.4 ± 9.8	0.292^a^
**Tumor Size [cm]**			
Mean ± SD ** ≤4** ** >4**	6.1 ± 2.9 4 (44.4%) 5 (55.6%)	5.82 ± 2.4 8 (42%) 11 (58%)	0.602^b^
**Tumor grade**			
G1 G2 G3 G4	0 (0%) 6 (66.7%) 2 (22.2%) 1 (11.1%)	0 (0%) 14 (73.7%) 5 (26.3%) 0 (0%)	0.334^b^
**Axillary Lymph Node Metastasis**			
Negative Positive	1 (11.1%) 8 (88.9%)	4 (21.1%) 15 (78.9%)	0.473^b^
**Lymphovascular invasion**			
Negative Positive	5 (55.6%) 4 (44.4%)	2 (10.5%) 17 (89.5%)	0.021^*b^
**ER**			
Negative Positive	6 (66.7%) 3 (33.3%)	12 (63.2%) 7 (36.8%)	0.601^b^
**PR**			
Negative Positive	6 (66.7%) 3 (33.3%)	11 (57.9%) 8 (42.1%)	0.493^b^
**HER-2**			
Negative Positive	7 (77.8%) 2 (22.2%)	11 (57.9%) 8 (42.1%)	0.278^b^
**Neoadjuvant Chemotherapy**			
Not received Received	4 (44.4%) 5 (55.6%)	7 (36.8%) 12 (63.2%)	0.507^b^

Data are reported as means ± SD.

a Student’s t-test.

b Chi-square test.

*Significant P value (p< 0.05).

### HCMV^+^ IBC Cancer Tissues Are Characterized by Significant High Infiltration of CD163^+^ and MAC387^+^ TAMs

CD14^+^ monocytes were found to be a reservoir for HCMV latency (18) and infected macrophages serve as “mobile vectors” for HCMV spreading and dissemination to breast tissue (17). In addition, we previously found high infiltration of CD14^+^ monocytes in IBC cancer tissues compared to non-IBC (64). There is also an increased incidence of CD68^+^ and CD163^+^ TAMs associated with breast cancer progression (65–67), including MAC387^+^ macrophages that are associated with viral infection (22). Thus, we determined the incidence of CD14^+^ monocytes, and CD68^+^, CD163^+^ and MAC387^+^ TAMs in the cancer tissues of HCMV^+^ IBC patients. Statistical analysis of IHC imaging data showed a significant (*P*= 0.04) infiltration of CD14^+^ monocytes in HCMV^+^ IBC cancer tissues compared to the HCMV^-^ tissues (**Figures 1E, F**). It was demonstrated that a high incidence of migratory TAMs correlates with aggressiveness and worse outcomes in breast cancer (68). We therefore determined the infiltration of CD68^+^ and MAC387^+^ TAMs in HCMV^-^ and HCMV^+^ cancer tissues of IBC patients. Statistical analysis showed no significant differences in the infiltration of CD68^+^ TAMs (**Figures 1G, H**) in HCMV+ compared to HCMV- IBC cancer tissues, while HCMV+ IBC cancer tissues were characterized by significant high infiltration of CD163^+^ (**Figures 1I, J**) and MAC387^+^ TAMs (**Figures 1K, L**) (P= 0.48 and 0.04, respectively) compared to HCMV- IBC cancer tissues.

### Microscopic Live-Cell Imaging and Gene Expression Signature of *In Vitro* Polarized HCMV^-^ and HCMV^+^ TAMs

**Figure 2:**
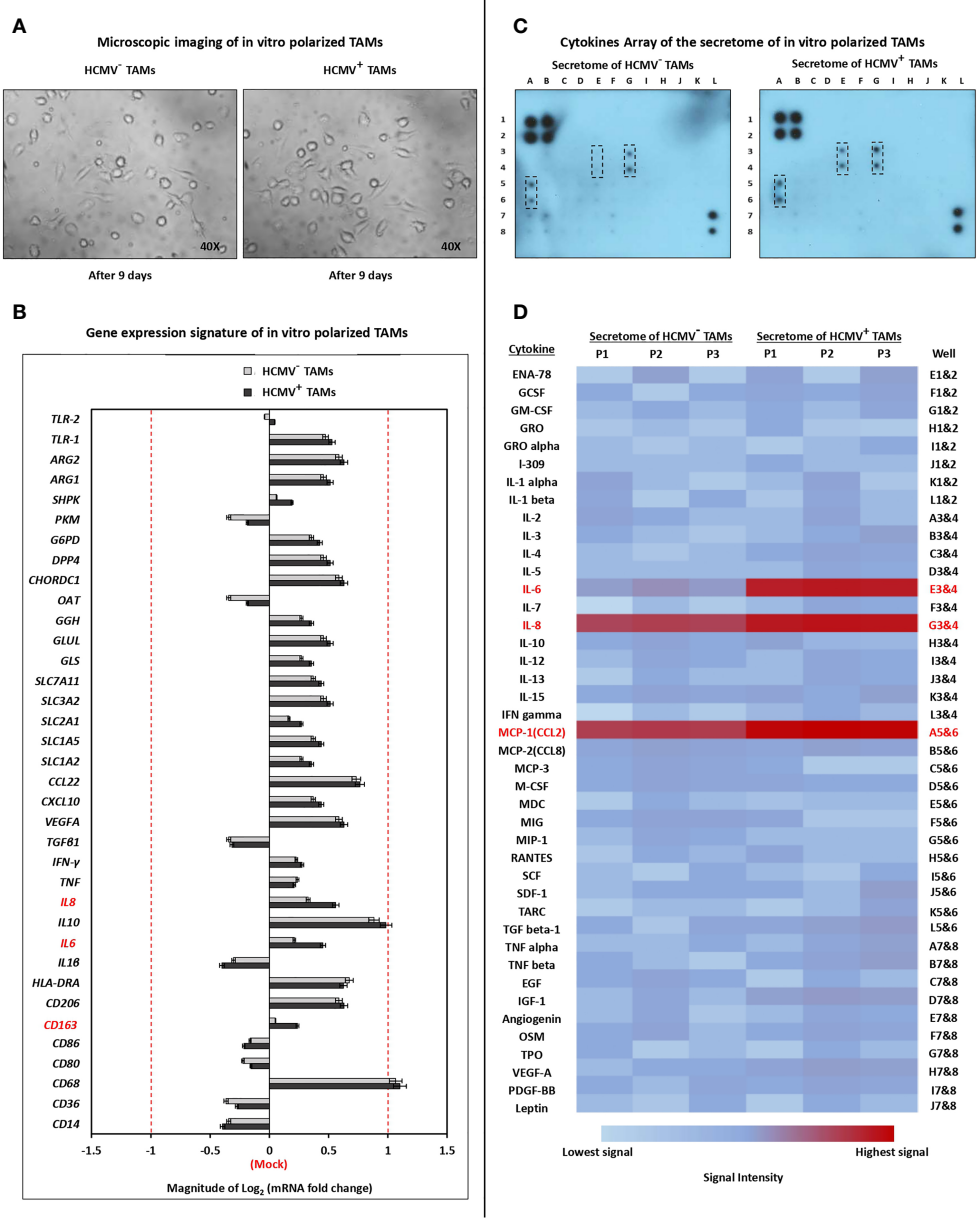
Characterization of polarized TAMs *via* microscopic examination, quantitative Real-time PCR, and cytokine array. **(A)** Microscopic images show polarization status of TME CD14^+^ monocytes isolated from HCMV^-^ and HCMV^+^ IBC patients. (M0) were identified as small/roundish cells without cytoplasmic projections on the cell surface, (M1) were identified as enlarged amoeboid cells characterized by presence of many delicate cytoplasmic extensions on the cell surface with visible intracellular vacuoles, and (M2) were identified as a large and elongated “spindeloid” cells with cytoplasmic extensions on the apical ends of the cell bodies. **(B)** Clustered bars represent the mRNA expression of macrophage polarization-related genes in HCMV^-^ and HCMV^+^ polarized TAMs compared to mock cells. **(C)** Membrane-based cytokine profiling array. Spots represent the signal intensity value of each cytokine, calculated using ImageJ software (NIH, Bethesda, MA, USA) and normalized according to an algorithm provided in the cytokine antibody array kit instruction manual. **(D)** Heat map showing expression levels of 42 cytokines in HCMV^-^ versus HCM^+^ polarized TAMs. Genes and cytokines in red color are significantly highly expressed in HCMV^+^ TAMs compared to HCMV^-^ TAMs. Data represented the mean of ± SD (number of experimental replicates = 3). *P* < 0.05 consider significant as calculated using Student *t*-test.

Microscopic examination showed that the secretome of SUM149 cells polarized isolated HCMV^-^ and HCMV^+^ TME CD14^+^ monocytes towards M1/M2 TAMs (**Figure 2A**). The gene expression signature showed that the secretome of SUM149 cells activates the polarization of both HCMV^-^ and HCMV^+^ TME CD14^+^ monocytes towards M1/M2 TAMs. Moreover, the mRNA level of *IL-6*, *IL-8* and *CD163* were significantly (*P* = 0.03, 0.03 and 0.04, respectively) more highly expressed in HCMV^+^ TAMs compared to HCMV^-^ TAMs (**Figure 2B**).

### Cytokine Profiling of the Secretome of HCMV^-^ and HCMV^+^ TAMs

The human cytokine array analysis showed that the secretome of HCMV^+^ TAMs is characterized by significantly higher expression level of IL-6 (*P*= 0.01), IL-8 (*P*= 0.03), and MCP-1 (*P*= 0.04) compared to the secretome of HCMV^-^ TAMs (**Figures 2C, D**).

### Secretome of HCMV^+^ TAMs Significantly Enhance Proliferation, Invasion, and Colony Formation of SUM149 Cells

**Figure 3:**
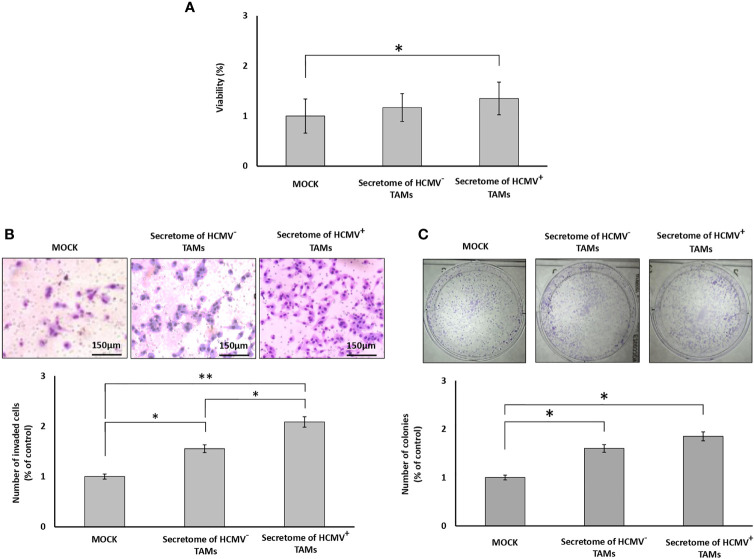
Secretome of HCMV^+^ TAMs enhance proliferation, invasion, and colony formation in SUM149 cells. **(A)** Bars show cell proliferation percentages of SUM149 cells stimulated by the secretome of HCMV^-^ and HCMV^+^ compared to mock cells. **(B)** Microscopic images of SUM149 cells that invaded the Coat Matrigel-coated lower chamber in response to the secretome of HCMV^-^ and HCMV^+^ TAMs compared to mock cells. Bars represent the percentage of invasive SUM149 cells in response to the secretome of HCMV^-^ and HCMV^+^ TAMs compared to mock cells. **(C)** Wells showed the clonogenic ability of SUM149 cells in response to secretome of HCMV^-^ and HCMV^+^ TAMs compared to mock cells. Bars represent the number of colonies of SUM149 cells in response to the secretome of HCMV^-^ and HCMV^+^ TAMs compared to mock cells. Data represented the mean of ± SD (number of experimental replicates = 3). *P* values were as determined by one-way ANOVA test followed by Tukey’s HSD and *Post Hoc* tests, where * represented (*P* < 0.05) and ** represented (*P* < 0.01).

MTT assay results showed a non-significant increase in the proliferation of SUM149 cells in the presence of the secretome of HCMV^-^ TAMs, while showing a significant increase (*P*= 0.04) in the proliferation of SUM149 cells in the presence of the secretome of HCMV^+^ TAMs compared to control cells (**Figure 3A**). To test whether the secretome of HCMV^-^ and HCMV^+^ TAMs promote the invasive properties of SUM149 cells *via* secretion of proteases, we used BD Matrigel invasion chambers. Invading cells are those that can degrade Matrigel *via* secretion of proteases and pass through the coated filters of Matrigel invasion chambers. Statistical analysis showed that the secretome of HCMV^-^ and HCMV^+^ TAMs significantly (*P*= 0.04 and 0.01, respectively) enhanced the invasive properties of SUM149 cells by 155.3% and 208.5%, respectively compared to control SUM149 cells (**Figure 3B**). In addition, clonogenic assay results revealed a significant increase in the colony formation potential of SUM149 cells seeded in presence of the secretome of both HCMV^-^ and HCMV^+^ TAMs (*P*= 0.04 and 0.03, respectively) compared to control cells. In contrast, there are no significant differences in the colony formation potential of SUM149 cells seeded in presence of the secretome of both HCMV^-^ and HCMV^+^ TAMs (**Figure 3C**).

### Secretome of HCMV^+^ TAMs Alters the Expression of Extracellular Matrix and Cell Adhesion Molecule Genes in SUM149 Cells: Functional Enrichment Analysis and PPI Network Construction

**Figure 4:**
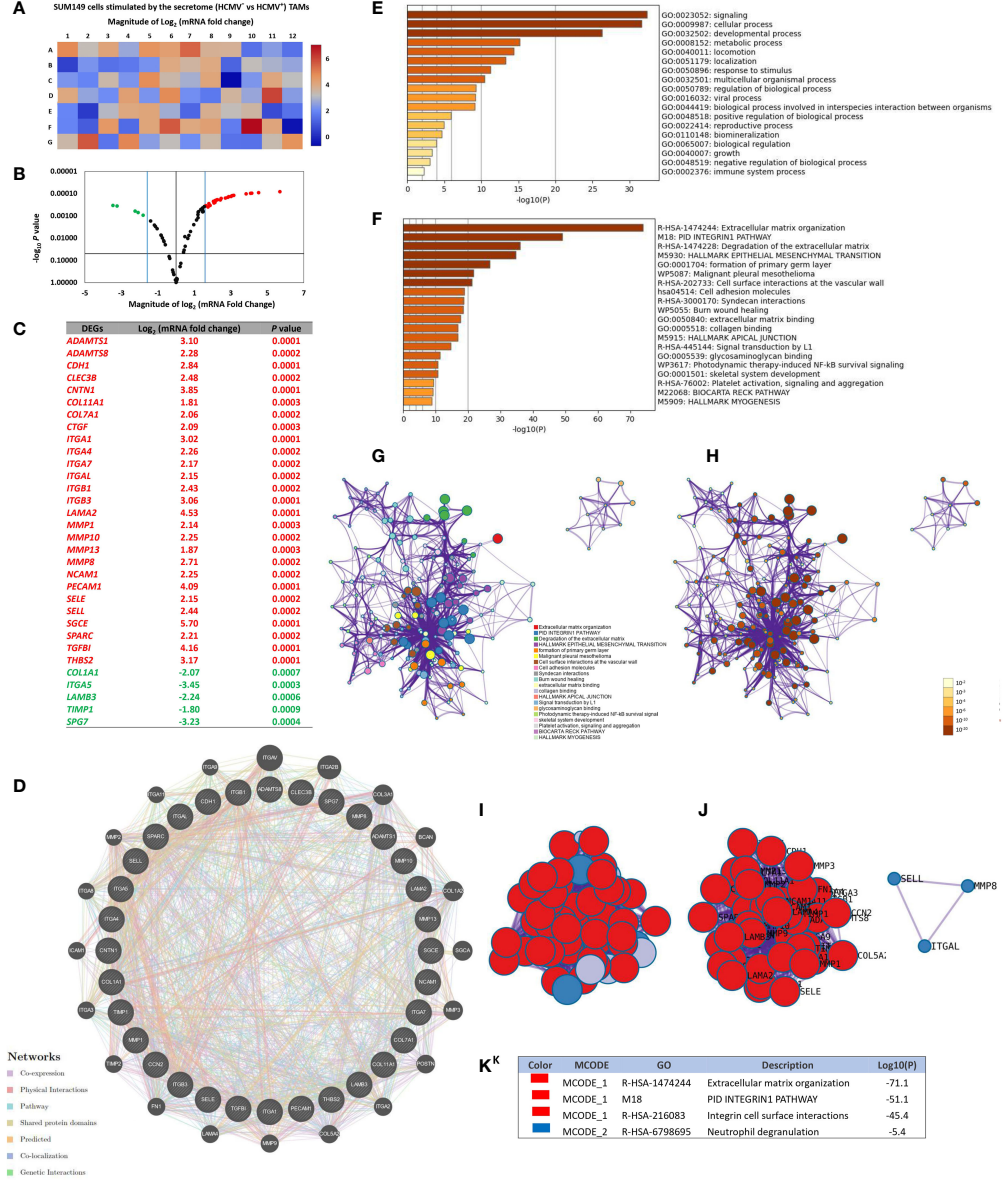
Up- and downregulated extracellular matrix and cell adhesion genes of SUM149 cells stimulated by the secretome of HCMV^+^ polarized TAMs versus HCMV^+^ polarized TAMs. **(A, B)** Heat map and volcano plot showing significant up- and downregulated genes in SUM149 cells stimulated by the secretome of HCMV^+^ polarized TAMs compared to HCMV^+^ polarized TAMs. **(C)** Table showing the Log2 mRNA fold change and significant *P* values of up- and downregulated genes in SUM149 cells stimulated by the secretome of HCMV^+^ polarized TAMs versus HCMV^+^ polarized TAMs. **(D)** Gene-gene interaction network of different expressed extracellular matrix and cell adhesion molecule genes and their neighboring genes. Each node represents a gene, and the size of which represents the strength of the interaction. **(E)** Bar graph of the top-level Gene Ontology biological processes, with *P*-values. **(F)** Bar graph of enriched terms across input gene lists, with *P* -values. **(G, H)** Network of enriched terms: colored by cluster ID, where nodes that share the same cluster ID are typically close to each other; colored by p-value, where terms containing more genes tend to have a more significant *P*-value. **(I, J)** Protein-protein interaction network and MCODE components identified in the gene list. **(K)** Table showed the functional analysis of MCODE1 and MCODE2 components.

RT^2^ extracellular matrix (ECM) and cell adhesion (CA) mRNA PCR array results showed that the CA mRNAs *CDH1, CNTN1, ITGA1, ITGA4, ITGA7, ITGAL, ITGB1, ITGB3, NCAM1, PECAM1, SELE, SELL* and *SGCE*, and ECM proteins *ADAMTS1, ADAMTS8, CLEC3B, COL11A1, COL7A1, CTGF, LAMA2, MMP1, MMP10, MMP13, MMP8, SPARC, TGFBI*, and *THBS2* were significant more highly expressed than other CA and ECM mRNAs in SUM149 cells stimulated by the secretome of HCMV^+^ TAMs, compared to SUM149 cells stimulated by the secretome of HCMV^-^ TAMs. On the other hand, the CA mRNAs *ITGA5* and those corresponding to ECM *COL1A1, SPG7, LAMB3*, and *TIMP1* were significantly more reduced in expression than other CA and ECM mRNAs in SUM149 cells stimulated by the secretome of HCMV^+^ TAMs compared to SUM149 cells stimulated by the secretome of HCMV^-^ TAMs. (**Figures 4A–C**).

Through GeneMANIA analysis, we identified the top 20 neighboring genes with the highest frequency association with differential expressed CA and ECM-related genes in SUM149 cells stimulated by the secretome of HCMV^+^ TAMs. These data indicate that *ITGAV*, *ITGA2B*, *COL3A1*, *BCAN*, *COL1A2*, *SGCA*, *MMP3*, *POSTN*, *ITGA2*, *COL5A2*, *MMP9*, *LAMA4*, *FN1*, *TIMP2*, *ITGA3*, *ICAM1*, *ITGA8*, *MMP2*, *ITGA11*, and *ITGA9* were associated with the function and pathway of differential expressed CA and ECM -related genes (**Figure 4D**). The functions of these CA and ECM -related genes and their neighboring genes were predicted using Metascape. The top 18 GO enrichment items of these CA and ECM -related genes and their neighboring genes were described in (**Figure 4E**), which mainly included signaling, cellular process, locomotion, localization, and viral processes. Pathway enrichment analysis represented pathways associated with IBC progression and metastasis including extracellular matrix organization, PID Integrin-1 pathway, degradation of the extracellular matrix, hallmark epithelial mesenchymal transition and cell adhesion molecules, involved in IBC, the top 20 associated pathways are described in (**Figure 4F**) and **Supplementary Table S3**. Moreover, to better understand the relationship between CA and ECM -related genes and their neighboring genes, and IBC, we then performed a Metascape protein-protein interaction (PPI) enrichment analysis. The PPI network and MCODE components are shown in (**Figures 4G–K**). Data showed that the biological functions of CA and ECM-related genes and their neighboring genes are mainly enriched in formation of Extracellular matrix organization, PID Integrin1 pathway, Integrin cell surface interactions, and Neutrophil degranulation in IBC.

### Secretome of HCMV+ TAMs Enhances the Expression of BCSC-Related Genes in SUM149 Cells: Functional Enrichment Analysis and PPI Network Construction

HCMV infection in the TME can induce stemness and an EMT program, leading to a potential increase in the progression of tumors including breast cancer (69–71). In addition, a higher incidence of CD133^+^/HCMV-IE^+^ cells is associated with poor patient survival (72). Herein, the BCSC-related gene PCR array results showed that the mRNA expression of 26 genes out of 43 genes was significantly (*P* < 0.05) differentially expressed (2-fold cut off) in both stimulated SUM149 cells compared to mock cells **Supplementary Data (Figure S1)**.

**Figure 5:**
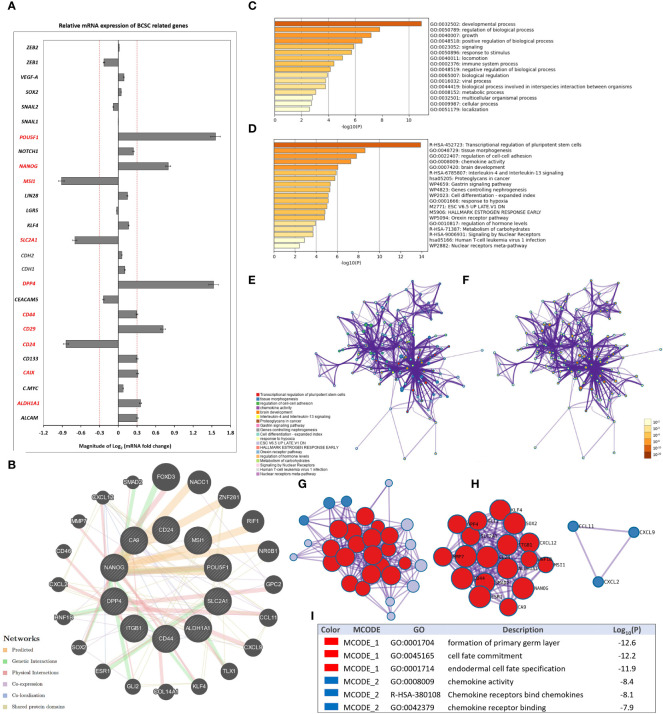
Secretome of HCMV^+^ TAMs enhance the expression of BCSC-related genes in SUM149 cells. **(A)** Clustered bars represent the mRNA expression of BCSC-related genes in SUM149 cells stimulated by the secretome of HCMV^-^ and HCMV^+^ TAMs compared to mock cells. Data represent the mean of ± SD (number of experimental replicates = 3). *P* < 0.05 consider significant as calculated using Student *t*-test. **(B)** Gene-gene interaction network of upregulated BCSC-related genes and their neighboring genes. Each node represents a gene, and the size of which represents the strength of the interaction. **(C)** Bar graph of the top-level Gene Ontology biological processes, colored by *P*-values. **(D)** Bar graph of enriched terms across input gene lists, colored by *P*-values. **(E, F)** Network of enriched terms: firstly colored by cluster ID, where nodes that share the same cluster ID are typically close to each other; secondly colored by p-value, where terms containing more genes tend to have a more significant *P*-value. **(G, H)** Protein-protein interaction network and MCODE components identified in the gene list. **(I)** Table showed the functional analysis of MCODE1 and MCODE2 components.

More precisely, SUM149 cells stimulated by the secretome of HCMV^+^ TAMs were characterized by significantly (*P* < 0.05) higher mRNA expression (2-fold cut off) of *POU5F1, NANOG*, MSI1, *SLC2A1*, *DPP4*, *CD44*, *CD133*, *CD29*, *CAIX*, and *ALDH1A1*, while significantly lower expression of *CD24* was found compared to SUM149 cells stimulated by the secretome of HCMV^-^ TAMs (**Figure 5A**).

GeneMANIA analysis identified the top 20 neighboring genes with the highest frequency association with differentially expressed BCSC-related genes in SUM149 cells stimulated by the secretome of HCMV^+^ TAMs. These data indicate that *FOXD3*, *NACC1*, *ZNF281*, *RIF1*, *NR0B1*, *GPC2*, *CCL11*, *CXCL9*, *TLX1*, *KLF4*, *COL14A1*, *GLI2*, *ESR1*, *SOX2*, *HNF1B*, *CXCL2*, *CD46*, *MMP7*, *CXCL12*, and *SMAD2* were associated with the function and pathway of differentially expressed BCSC-related genes (**Figure 5B**). The functions of these BSCS genes and their neighboring genes were predicted using Metascape. The top 16 GO enrichment items of those upregulated BSCS-related genes and their neighboring genes described in (**Figure 5C**), which mainly included developmental process, regulation of biological process, signaling, response to stimulus, locomotion, immune system process, and viral process. Pathway enrichment analysis represented pathways associated with IBC progression and stemness including transcriptional regulation of pluripotent stem cells, tissue morphogenesis, chemokine activity, Interleukin-4 and Interleukin-13 signaling, and proteoglycans in cancer, the top 19 associated pathways are described in (**Figure 5D**) and **Supplementary Table S4**. Moreover, to better understand the relationship between differentially expressed BSCS-related genes with their neighboring genes, and IBC, we performed a Metascape protein-protein interaction (PPI) enrichment analysis. The PPI network and MCODE components are shown in (**Figures 5E–I**). Data showed that the biological functions of upregulated BSCS-related genes and their neighboring genes are mainly enriched in formation of primary germ layer, cell fate commitment, endodermal cell fate specification, chemokine activity, chemokine receptors bind chemokines, and chemokine receptor binding in IBC.

### Secretome of HCMV^+^ TAMs Induce Activation of p-STAT3, p-AMPKα, p-PRAS40, and p-SAPK/JNK Intracellular Signaling Molecules in SUM149 Cells

**Figure 6:**
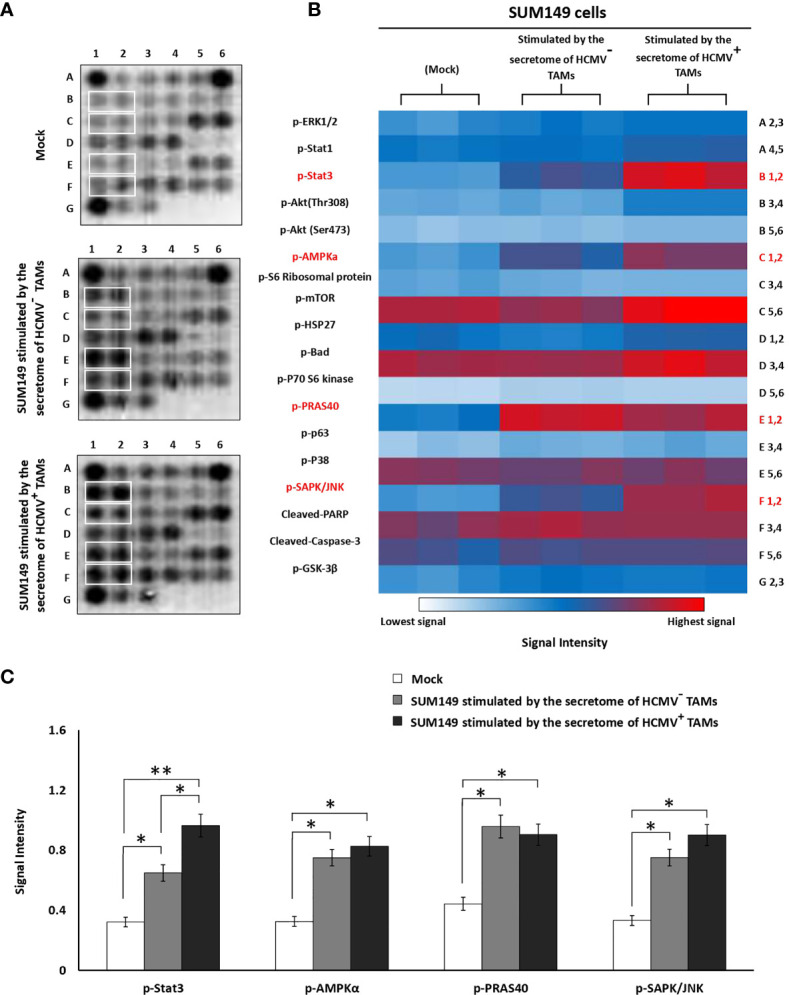
Intracellular pathways array analysis of SUM149 cells stimulated by the secretome of HCMV^-^ and HCMV^+^ TAMs compared to mock cells. **(A)** PathScan^®^ intracellular array membranes of SUM149 cells stimulated by the secretome of HCMV^-^ and HCMV^+^ TAMs compared to mock cells. **(B)** Heat map showing the different activation levels of eighteen signaling molecules in SUM149 cells stimulated by the secretome of HCMV^-^ and HCMV^+^ TAMs compared to mock cells. **(C)** Bars represent a high expression of p-STAT3, p-AMPKa, p-PRAS40, and p-SAPK/JNK intracellular cell signaling molecules in SUM149 cells stimulated by the secretome of HCMV^-^ and HCMV^+^ TAMs compared to mock cells. * Represented (*P* < 0.05) and ** represented (*P* < 0.01) as determined by one-way ANOVA test followed by Tukey’s HSD and *Post Hoc* tests. Data represented as means ± SD (number of experimental replicates = 3).

To further investigate whether the secretome of HCMV^+^ TAMs may alter intracellular signaling pathways of SUM149 cells, we used a PathScan Intracellular Signaling Array that detects 18 phosphorylated or cleaved signaling molecules per test. We measured changes in the expression of signaling molecules by SUM149 cells before and after stimulation by the secretome of HCMV^-^ and HCMV^+^ TAMs (**Figure 6A**). Statistical analysis revealed that secretome of HCMV^-^ and HCMV^+^ TAMs significantly upregulated p-STAT3 (*P*= 0.03 and 0.01, respectively), p-AMPKα (*P*= 0.04 and 0.03, respectively), p-PRAS40 (*P*= 0.03 and 0.04, respectively) and p-SAPK/JNK (*P*= 0.04 and 0.03, respectively) signaling molecules compared to control SUM149 cells. Moreover, statistical analysis revealed that secretome of HCMV^+^ TAMs significantly upregulated p-STAT3 (*P*= 0.04) in stimulated SUM149 cells compared to secretome of HCMV^-^ TAMs (**Figures 6B, C**).

## Discussion

The DNA and proteins of several onco-modulatory viruses have been found in breast cancers and associated with the disease initiation process although causality has not been established (73). HCMV is an onco-modulatory virus was found in more than 90% of primary and metastatic breast cancers (71). In addition, increased expression of HCMV proteins in breast cancer tissues was correlated with more aggressive breast cancer phenotypes (14, 71, 74, 75). HCMV is also known to promote a cellular secretome of several immunosuppressive or tumor-promoting mediators that affect the tumor microenvironment (76). These include the expression of transforming growth factor β (TGF-β) and IL-10 by T regulatory (Treg) cells, which inhibit the functions of natural killer (NK) cells and T cells and promote the EMT (77, 78). Furthermore, HCMV produces its immunosuppressive protein cmvIL-10, encoded by the *UL111A* gene, which induces maturation of pro-tumoral M2 macrophages and expression of the proto-oncogene Bcl-3 (77). cmvIL-10, UL37/vMIA, pUL144 which is a tumor necrosis factor (TNF) receptor homolog, and pUL128, a CC-like chemokine encoded by HCMV, modulate monocyte activity (79). In glioblastoma, HCMV infection confers glioblastoma stem cell (GSC) properties (80). The interplay of cancer cell stemness with commonly detected HCMV infection is poorly understood in medulloblastomas, where HCMV infection induces stem cell properties in the TMA (81). In this regard, Chan and colleagues suggested that HCMV stimulates monocyte reprogramming into M1/M2 macrophage phenotype *via* activation of NF-κB and P13K signaling pathway (82).

HCMV induces STAT3, TGF-β, IL-10 and cmvIL-10 in polarized M2 macrophages, leading to an immunosuppressive phenotype that is closely associated with TAMs (77, 82). Thus, HCMV infection of breast epithelial cells may contribute to neoplastic transformation and differentiation of M2-macrophages into the same tissue compartment. *In vitro* studies using different cancer cell lines revealed that HCMV infection in the TME may induce stemness and EMT in cancer cells, leading to increased cancer aggressiveness (72). Of note, EMT has a complex association with increased cancer stem cell properties that facilitates cancer cell motility and invasion (83). Thus, our results agree with Zhu and colleagues indicating that HCMV infection can induce EMT and stem cell properties of cancer cells (glioblastoma), in turn promoting increased cancer cell invasion and dissemination, through a mechanism that involves increased activity of JNK pathway (84). Moreover, latent CMV infection in a mouse xenograft tumor model, showed an increase in cell proliferation in number of lung metastatic nodules (69). In the spontaneous HCMV mouse glioblastoma model, the immediate-early (IE) proteins also augment stem cell properties in cancer cells (85).

There are therefore a number of ways in which HCMV can promote greater malignancy and BCSC activity. In breast cancer, HCMV^+^ TAMs secrete CCL18, which promotes the EMT of breast cancer cells, leading to increased metastasis (73, 86). Our previous studies showed an association between the incidence of HCMV DNA and IBC poor prognosis (7–9), but the potential onco-modulatory effect of HCMV in IBC is still not clearly understood. Here, we determined the prevalence of CD163^+^ and MAC387^+^ TAMs in the cancer tissues of HCMV^+^ compared to HCMV^-^ IBC patients. In addition, we tested the effect of the secretome of HCMV^+^ TAMs compared to HCMV^-^ TAMs on the proliferation, invasion, colony formation, and expression of BCSC-related genes in IBC cell line SUM149.

Previously, we found that CD14^+^ cells highly infiltrate the tumor microenvironment of IBC compared to non-IBC patients (9). In our present study, IHC analysis showed a significantly high infiltration of CD14^+^ monocytes in HCMV^+^ IBC cancer tissues compared to the HCMV^-^ tissues. During HCMV primary infection, HCMV^+^ monocytes migrate into tissues and differentiate into macrophages (87). HCMV-infected macrophages generate an appropriate microenvironment for virus replication, and these macrophages have a high survival rate (87, 88). Infected macrophages serve as “mobile vectors” for virus spreading and dissemination to different organs mainly by transendothelial migration (17). HCMV-infected macrophages show classical macrophage markers but less phagocytic and immunogenic capacity (89). Furthermore, HCMV^+^ IBC cancer tissues are characterized by significantly high infiltration of CD163^+^ and MAC387^+^ TAMs in comparison with HCMV^-^ IBC cancer tissues. High infiltration of CD163^+^ TAMs are associated with poor survival in breast cancer patients (90). Patients with CD163^+^ cancer cells are characterized by shorter disease-free survival after radiotherapy (91). CD163^+^ cancer cells were suggested to be caused *via* fusion between TAMs and cancer cells (92, 93). It was demonstrated that a high incidence of migratory MAC387^+^ TAMs correlated with aggressiveness and worse outcomes in breast cancer (68). MAC387^+^ TAMs were associated with high-grade, HR-negative breast cancers and early recurrence (68, 94, 95). It was demonstrated that MAC387 was expressed by a subset of TAMs as well as by a subset of cancer cells as a result of the fusion between TAMs and cancer cells leading to increase disease aggressiveness (96–98). HCMV infects macrophages and induces M1/M2 phenotype, close to the TAMs, which is associated with breast cancer poor prognosis (15). For instance, HCMV-DB clinical isolates trigger M2 polarization, and upregulates the proto-oncogene Bcl-3 (99). Our gene expression signature analysis showed that the secretome of SUM149 cells activates the polarization of both HCMV^-^ and HCMV^+^ TME CD14^+^ monocytes towards TAMs. Moreover, the mRNA levels of *IL-6*, *IL-8*, and *CD163* were significant highly expressed in HCMV^+^ TAMs compared to HCMV^-^ TAMs. In addition, our human cytokine array analysis showed that the secretome of HCMV^+^ TAMs is characterized by the significantly higher expression level of IL-6, IL-8, and MCP-1/CCL2 compared to the secretome of HCMV^-^ TAMs. IL-6 and IL-8 are cytokines associated with BCSCs and treatment resistance in breast cancer patients (100). Benner and colleagues demonstrated that TAMs stimulated by conditioned media of MDA-MB-231 cells displayed an increase in the co-expression of CD163/CD206 compared to *in vitro* M2- like macrophages (101). Indeed, these generated *in vitro* TAMs exhibited high transcriptional levels of IL-6, IL-10, CCL2, c-Myc, iNOS, and arginase compared to *in vitro* M2-like macrophages (101). Reinartz and colleagues showed that TAMs significantly express high M2 marker mRNA levels of *CD163*, *IL10*, and *VEGFA* compared to monocyte-derived macrophages (MDMs). In contrast, TAMs significantly express high M2 marker mRNA levels of *IL27RA*, *CCL18*, *CCL22* and *MMP9* compared to MDMs. In addition, TAMs significantly express high levels of several M1 markers such as *CD86*, *CCR2*, and *TNF* relative to MDMs (102). Our results showed that the secretome of HCMV^+^ TAMs enhances cell proliferation, invasion, colony formation, and alters the expression of many CA and ECM mRNAs. In agreement with our results, it was demonstrated that TAMs have potential roles in cancer cell proliferation (103), invasion (104), angiogenesis (105), and metastasis (106). EGF, FGFs, and VEGFs secreted by TAMs promote cancer cell proliferation, and angiogenesis (107, 108).

HCMV infection enhances cell proliferation, migration, and upregulation of EMT markers in colorectal cancer-derived stem cell-like cells (109). In line with our findings, HCMV infection in the TME can induce a cancer cell EMT program, resulting in increasing the aggressiveness of cancers (72). Fiallos and colleagues revealed that long-term HCMV infection promotes cell proliferation in glioma stem-like cells (GSC) (110).Moreover, cmvIL-10 facilitates cell migration and invasion *via* upregulation of both urokinase plasminogen receptor (uPAR) and plasminogen activator inhibitor-1 (PAI-1), which can stimulate MMP-3 activity in MDA-MB-231 cells (111). Moreover, HCMV infection alters the MMP-9/TIMP-1 balance in macrophages through immediate-early (IE) gene and late viral gene expression (112). Herein, SUM149 cells stimulated by the secretome of HCMV^+^ TAMs are characterized by the significantly high mRNA expression level of 10 BCSC-related genes as described in (**Figure 5A**) compared to SUM149 cells that are stimulated by the secretome of HCMV¯ TAMs.

We used gene enrichment analysis to identify the neighboring genes and the main functions and pathways of the differential expressed CA and ECM molecules and BCSC-related genes. The pathway enrichment and PPI network analysis of these differential expressed genes and their neighboring genes showed strong association mainly with degradation of the extracellular matrix, hallmark epithelial mesenchymal transition and cell adhesion molecules, transcriptional regulation of pluripotent stem cells, tissue morphogenesis, chemokine activity, Interleukin-4 and Interleukin-13 signaling, and proteoglycans in cancer. In agreement with previous studies (113–119), this bioinformatic analysis support our results which indicate a strong association between incidence of HCMV^+^ TAMs and IBC stemness properties and progression.

The results of PathScan Intracellular Signaling Array revealed that the secretome of HCMV^+^ TAMs significantly activated p-STAT3, p-AMPKα, p-PRAS40, and p-SAPK/JNK intracellular signaling molecules in SUM149 cells compared to mock cells. Furthermore, p-STAT3 significantly upregulated in SUM149 cells stimulated by the secretome of HCMV^+^ TAMs compared to SUM149 cells stimulated by the secretome of HCMV^-^ TAMs. Polyak and colleagues showed that in samples from IBC patients 40% of CD44^+^/CD24^-^ cells were positive for p-STAT3. In addition, they demonstrated that the inhibition of JAK2 decreases the proliferation rate of p-STAT3^+^ IBC cells *in vitro* and *in vivo* (120). AMP-activated protein kinase (AMPK) promotes EMT in breast cancer cells *via* Twist1 upregulation (121). Phospho-PRAS40 upregulates and activates EMT-related factors to induce metastasis *via* the phosphorylation of smad (122). JNK activity promotes invasion, and EMT in breast cancer cells *via* ERK activation (123). IL-6 and IL-8 secreted from adjacent cells in the TME such as TAMs activate the STAT3 signaling pathway in breast cancer cells (124). It is demonstrated that IL-6 is significantly expressed in IBC compared to non-IBC patients (125, 126). It was found that M2 macrophage-educated mesenchymal stem/stromal cells highly expressed IL-6 and increased IBC cell invasion and mammosphere formation (127). Moreover, a decrease in the infiltration of TAMs downregulates p-STAT3 and IL-6 secretion within the IBC TME, leading to reduced skin invasion and local recurrence (127). IBC cells highly expressed IL8 and growth-regulated oncogene (GRO) chemokines that activate the STAT3 signaling pathway, which promotes the EMT program. In addition, IBC cells attract and differentiate monocytes into TAMs, which were found to secrete high levels of IL8 and GRO chemokines that further promote the IBC EMT program (115).

## Conclusion

The secretome of HCMV^+^ TAMs increases the proliferation, invasion, colony formation, and increases expression of CA and ECM mRNAs and BCSC-related genes compared to mock cells by acting on p-STAT3, p-AMPKα, p-PRAS40, and p-SAPK/JNK intracellular signaling molecules. Our results highlight the critical onco-modulatory role of HCMV infection in IBC. Further studies are necessary to begin to establish whether there is causality in these associations, and to identify the clinical significance of our findings. A potential combination therapy would be to use anti-HCMV drugs with chemotherapy to improve the effectiveness of chemotherapy and decrease the resistance of cancer cells.

## Data Availability Statement

The original contributions presented in the study are included in the article/**Supplementary Material**, further inquiries can be directed to the corresponding author/s.

## Ethics Statement

The study protocol was reviewed and approved by the Institutional Review Board (IRB#00006379), Faculty of Medicine, Ain Shams University, Egypt. Before participation, all patients signed written informed consent forms, including approval for publication of the study results.

## Author Contributions

RJS and MMM applied for funding and directed the project. HTM and MMM suggested the idea and designed the research strategy and experimental protocols. Surgeon MES was responsible for enrolling patients. HTM collected patients’ clinical and pathological data. HTM and AAE conducted all practical experiments of the study. HTM analyzed the data using Statistical Package of the Social Sciences and biomedical informatics software. HTM, RJS, and MMM drafted and wrote the manuscript with the input of all co-authors.

## Funding

The study was supported by Avon Foundation Grants # 02-2009-085 a and b (RJS and MMM) and the Cairo University Scientific Research Sector (MMM), and Breast Cancer Research Foundation (BCRF 18-146) (RJS).

## Conflict of Interest

The authors declare that the research was conducted in the absence of any commercial or financial relationships that could be construed as a potential conflict of interest.

## Publisher’s Note

All claims expressed in this article are solely those of the authors and do not necessarily represent those of their affiliated organizations, or those of the publisher, the editors and the reviewers. Any product that may be evaluated in this article, or claim that may be made by its manufacturer, is not guaranteed or endorsed by the publisher.
