# Supplementary material for: Inflammatory Breast Cancer: The Secretome of HCMV+ Tumor-Associated Macrophages Enhances Proliferation, Invasion, Colony Formation, and Expression of Cancer Stem Cell Markers
Source: Front Oncol. 2022 Jun 30;12:899622. doi: 10.3389/fonc.2022.899622 (PMC9281473; doi:10.3389/fonc.2022.899622)
Supplement: Supplementary file 1 [file DataSheet_1.docx]

**Supplementary tables:**

**Table S1**: List of oligonucleotide primers of macrophages polarization-related genes array

| **Gene** | **Forward Primer** | **Reverse Primer** |
| --- | --- | --- |
| *ARG1* | 5'-GTGGAAACTTGCATGGACAAC-3' | 5'-AATCCTGGCACATCGGGAATC-3' |
| *ARG2* | 5'-CGCGAGTGCATTCCATCCT-3' | 5'-TCCAAAGTCTTTTAGGTGGCAG-3' |
| *CCL2* | 5'-CAGCCAGATGCAATCAATGCC-3' | 5'-TGGAATCCTGAACCCACTTCT-3' |
| *CCL22* | 5'-ATCGCCTACAGACTGCACTC-3' | 5'-GACGGTAACGGACGTAATCAC-3' |
| *CD11c* | 5'-AGAGCTGTGATAAGCCAGTTCC-3' | 5'-AATTCCTCGAAAGTGAAGTGTGT-3' |
| *CD14* | 5'-AGCCAAGGCAGTTTGAGTCC-3' | 5'-TAAAGGACTGCCAGCCAAGC-3' |
| *CD163* | 5'-CAGGAAACCAGTCCCAAACA-3' | 5'-AGCGACCTCCTCCATTTACC-3' |
| *CD204* | 5'-GCAGTGGGATCACTTTCACAA-3' | 5'-AGCTGTCATTGAGCGAGCATC-3' |
| *CD206* | 5'-TTCGGACACCCATCGGAATTT-3' | 5'-CACAAGCGCTGCGTGGAT-3' |
| *CD36* | 5'-GCCAAGGAAAATGTAACCCAGG-3' | 5'-GCCTCTGTTCCAACTGATAGTGA-3' |
| *CD68* | 5'-GCTACATGGCGGTGGAGTACAA-3' | 5'-ATGATGAGAGGCAGCAAGATGG-3' |
| *CD80* | 5'-AAACTCGCATCTACTGGCAAA-3' | 5'-GGTTCTTGTACTCGGGCCATA-3' |
| *CD86* | 5'-CTGCTCATCTATACACGGTTACC-3' | 5'-GGAAACGTCGTACAGTTCTGTG-3' |
| *CHORDC1* | 5'-CCTTGCTGTGCTACAACCG-3' | 5'-CGGAACACCTGGGTGGTATG-3' |
| *CXCL10* | 5'-CGCTGTACCTGCATCAGCATTAG-3' | 5'-CTGGATTCAGACATCTCTTCTCACC-3' |
| *DPP4* | 5'-GGGTCACATGGTCACCAGTG-3' | 5'-TCTGTGTCGTTAAATTGGGCATA-3' |
| *G6PD* | 5'-CGAGGCCGTCACCAAGAAC-3' | 5'-GTAGTGGTCGATGCGGTAGA-3' |
| *GGH* | 5'-GGCTGGATCTTACAGAGAAAGAC-3' | 5'-ACTCTCCACTAATCAGCAGTGA-3' |
| *GLS* | 5'-AGGGTCTGTTACCTAGCTTGG-3' | 5'-ACGTTCGCAATCCTGTAGATTT-3' |
| *GLUL* | 5'-AAGAGTTGCCTGAGTGGAATTTC-3' | 5'-AGCTTGTTAGGGTCCTTACGG-3' |
| *HLA-DRA* | 5'-AGTCCCTGTGCTAGGATTTTTCA-3' | 5'-ACATAAACTCGCCTGATTGGTC-3' |
| *IFN-γ* | 5'-TGGCTTTTCAGCTCTGCATC-3' | 5'-CCGCTACATCTGAATGACCTG-3' |
| *IL10* | 5’-TCAAGGCGCATGTGAACTCC-3’ | 5’-GATGTCAAACTCACTCATGGCT-3’ |
| *IL12A* | 5'-CCTTGCACTTCTGAAGAGATTGA-3' | 5'-ACAGGGCCATCATAAAAGAGGT-3' |
| *IL1β* | 5'-AGCTACGAATCTCCGACCAC-3' | 5'-CGTTATCCCATGTGTCGAAGAA-3' |
| *IL23A* | 5'-CTCAGGGACAACAGTCAGTTC-3' | 5'-ACAGGGCTATCAGGGAGCA-3' |
| *IL6* | 5'-ACTCACCTCTTCAGAACGAATTG-3' | 5'-CCATCTTTGGAAGGTTCAGGTTG-3' |
| *IL8* | 5'-ATGACTTCCAAGCTGGCCGTGGCT-3' | 5'-TCTCAGCCCTCTTCAAAAACTTCTC-3' |
| *iNOS* | 5'-CGGTGCTGTATTTCCTTACGAGGCGAAGAAGG-3' | 5'-GGTGCTGCTTGTTAGGAGGTCAAGTAAAGGGC-3' |
| *OAT* | 5'-GTGGGGCTATACCGTGAAGG-3' | 5'-TGGTCCAAAACCATCGTAACTG-3' |
| *PKM* | 5'-ATGTCGAAGCCCCATAGTGAA-3' | 5'-TGGGTGGTGAATCAATGTCCA-3' |
| *SHPK* | 5'-CAAGCCCTACACGAGTGCC-3' | 5'-ACACCGGGGTAATCCCTCC-3' |
| *SLC1A2* | 5'-CCTGACGGTGTTTGGTGTCAT-3' | 5'-CAAGCGGCCACTAGCCTTAG-3' |
| *SLC1A5* | 5'-GAGCTGCTTATCCGCTTCTTC-3' | 5'-GGGGCGTACCACATGATCC-3' |
| *SLC2A1* | 5'-GGCCAAGAGTGTGCTAAAGAA-3' | 5'-ACAGCGTTGATGCCAGACAG-3' |
| *SLC3A2* | 5'-TGAATGAGTTAGAGCCCGAGA-3' | 5'-GTCTTCCGCCACCTTGATCTT-3' |
| *SLC7A11* | 5'-TCTCCAAAGGAGGTTACCTGC-3' | 5'-AGACTCCCCTCAGTAAAGTGAC-3' |
| *STAT1* | 5'-ATCAGGCTCAGTCGGGGAATA-3' | 5'-TGGTCTCGTGTTCTCTGTTCT-3' |
| *TGFβ1* | 5′-GCAGCACGTGGAGCTGTA-3′ | 5′-CAGCCGGTTGCTGAGGTA-3′ |
| *TLR-1* | 5'-TTCAAACGTGAAGCTACAGGG-3' | 5'-CCGAACACATCGCTGACAACT-3' |
| *TLR-2* | 5'-ATCCTCCAATCAGGCTTCTCT-3' | 5'-GGACAGGTCAAGGCTTTTTACA-3' |
| *TNF* | 5'-GAGGCCAAGCCCTGGTATG-3' | 5'-CGGGCCGATTGATCTCAGC-3' |
| *VEGFA* | 5'-AGGGCAGAATCATCACGAAGT-3' | 5'-AGGGTCTCGATTGGATGGCA-3' |
| *18S* | 5'-GGATGTAAAGGATGGAAAATACA-3' | 5'-TCCAGGTCTTCACGGAGCTTGTT-3' |
| *ACTB* | 5'-CATGTACGTTGCTATCCAGGC-3' | 5'-CTCCTTAATGTCACGCACGAT-3' |
| *B2M* | 5'-GAGGCTATCCAGCGTACTCCA-3' | 5'-CGGCAGGCATACTCATCTTTT-3' |
| *GAPDH* | 5'-GACAGTCAGCCGCATCTTCT-3' | 5'-TTAAAAGCAGCCCTGGTGAC-3' |
| *HPRT* | 5'-CAGTCCCAGCGTCGTGATTA-3' | 5'-TGGCCTCCCATCTCCTTCAT-3' |

**Table S2**: List of oligonucleotide primers of BCSC-related genes array

| **Gene** | **Forward Primer** | **Reverse Primer** |
| --- | --- | --- |
| *ALCAM* | 5`-ACTTGACGTACCTCAGAATCTCA-3` | 5`-CATCGTCGTACTGCACACTTT-3` |
| *ALDH1A1* | 5`-GCACGCCAGACTTACCTGTC-3` | 5`-CCTCCTCAGTTGCAGGATTAAAG-3` |
| *BMI-1* | 5`-CCACCTGATGTGTGTGCTTTG-3` | 5`-TTCAGTAGTGGTCTGGTCTTGT-3` |
| *BMP7* | 5`-TCGGCACCCATGTTCATGC-3` | 5`-GAGGAAATGGCTATCTTGCAGG-3` |
| *CAIX* | 5`-TTTGCCAGAGTTGACGAGGC-3` | 5`-GCTCATAGGCACTGTTTTCTTCC-3` |
| *CD133* | 5`-TTCTTGACCGACTGAGACCCA-3` | 5`-TCATGTTCTCCAACGCCTCTT-3` |
| *CD24* | 5`-CTCCTACCCACGCAGATTTATTC-3` | 5`-AGAGTGAGACCACGAAGAGAC-3` |
| *CD29* | 5`-CAAGAGAGCTGAAGACTATCCCA-3` | 5`-TGAAGTCCGAAGTAATCCTCCT-3` |
| *CD34* | 5`-ACCAGAGCTATTCCCAAAAGACC-3` | 5`-TGCGGCGATTCATCAGGAAAT-3` |
| *CD38* | 5`-CAACTCTGTCTTGGCGTCAGT-3` | 5`-CCCATACACTTTGGCAGTCTACA-3` |
| *CD44* | 5`-CTGCCGCTTTGCAGGTGTA-3` | 5`-CATTGTGGGCAAGGTGCTATT-3` |
| *CDH1* | 5`-ATTTTTCCCTCGACACCCGAT-3` | 5`-TCCCAGGCGTAGACCAAGA-3` |
| *CDH2* | 5`-CCATCAAGCCTGTGGGAATC-3` | 5`-GCAGATCGGACCGGATACTG-3` |
| *CEACAM5* | 5`-GACGCAAGAGCCTATGTATG-3` | 5`-GGCATAGGTCCCGTTATTA-3` |
| *c-MYC* | 5`-CACCAGCAGCGACTCTGA-3` | 5`-GATCCAGACTCTGACCTTTTGC-3` |
| *DPP4* | 5`-AGTGGCACGGCAACACATT-3` | 5`-AGAGCTTCTATCCCGATGACTT-3` |
| *GATA3* | 5`-GCCCCTCATTAAGCCCAAG-3` | 5`-TTGTGGTGGTCTGACAGTTCG-3` |
| *GATA4* | 5`-GTGTCCCAGACGTTCTCAGTC-3` | 5`-GGGAGACGCATAGCCTTGT-3` |
| *GLUT1* | 5`-AACTCTTCAGCCAGGGTCCAC-3` | 5`-CACAGTGAAGATGATGAAGAC-3` |
| *ITGA1* | 5`-CTGGACATAGTCATAGTGCTGGA-3` | 5`-ACCTGTGTCTGTTTAGGACCA-3` |
| *ITGA2* | 5`-CCTACAATGTTGGTCTCCCAGA-3` | 5`-AGTAACCAGTTGCCTTTTGGATT-3` |
| *ITGA4* | 5`-CACAACACGCTGTTCGGCTA-3` | 5`-CGATCCTGCATCTGTAAATCGC-3` |
| *KLF17* | 5`-GCTGCCCAGGATAACGAGAAC-3` | 5`-ATCTCTGCGCTGTGAGGAAAG-3` |
| *KLF4* | 5`-ATT GGACCCGGT GTACATTC-3` | 5`-AGCACGAAC TTGCCCATC-3` |
| *LGR5* | 5`-CACCTCCTACCTAGACCTCAGT-3` | 5`-CGCAAGACGTAACTCCTCCAG-3` |
| *LIN28* | 5`-CCAGTGGATGTCTTTGTGCA-3` | 5`-ACCCTTGGCTGACTTCTTAAA-3` |
| *MSI1* | 5`-TAAAGTGCTGGCGCAATCG-3` | 5`-TCTTCTTCGTTCGAGTCACCA-3` |
| *NANOG* | 5`-TCTCCTCTTCCTTCCTCCAT-3` | 5`-CCTTGTCTTCCTTTTTTGCG-3` |
| *NOS2* | 5`-AGGGACAAGCCTACCCCTC-3` | 5`-CTCATCTCCCGTCAGTTGGT-3` |
| *NOTCH1* | 5`-GAGGCGTGGCAGACTATGC-3` | 5`-CTTGTACTCCGTCAGCGTGA-3` |
| *NOTCH2* | 5`-CAACCGCAATGGAGGCTATG-3` | 5`-GCGAAGGCACAATCATCAATGTT-3` |
| *OCT3/4* | 5`-AGCAAAACCCGGAGGAGT-3` | 5`-CCACATCGGCCTGTGTATATC-3` |
| *SNAIL1* | 5`-ACTGCAACAAGGAATACCTCAG-3` | 5`-GCACTGGTACTTCTTGACATCTG-3` |
| *SNAIL2* | 5`-CGAACTGGACACACATACAGTG-3` | 5`-CTGAGGATCTCTGGTTGTGGT-3` |
| *SOX2* | 5`-CTCCGGGACATGATCAGC-3` | 5`-CTGGGACATGTGAAGTCTGC-3` |
| *TWIST1* | 5`-GTCCGCAGTCTTACGAGGAG-3` | 5`-GCTTGAGGGTCTGAATCTTGCT-3` |
| *TWIST2* | 5`-AGCGACGAGATGGACAATAAGATGACC-3` | 5`-CGGTCCGGAGGTGGGTGGCG-3` |
| *VEGF-A* | 5`-AGGGCAGAATCATCACGAAGT-3` | 5`-AGGGTCTCGATTGGATGGCA-3` |
| *VIM* | 5`-AGTCCACTGAGTACCGGAGAC-3` | 5`-CATTTCACGCATCTGGCGTTC-3` |
| *WEE1* | 5`-AACAAGGATCTCCAGTCCACA-3` | 5`-GGGCAAGCGCAAAAATATCTG-3` |
| *WNT1* | 5`-CGATGGTGGGGTATTGTGAAC-3` | 5`-CCGGATTTTGGCGTATCAGAC-3` |
| *ZEB1* | 5`-CAGCTTGATACCTGTGAATGGG-3` | 5`-TATCTGTGGTCGTGTGGGACT-3` |
| *ZEB2* | 5`-GGAGACGAGTCCAGCTAGTGT-3` | 5`-CCACTCCACCCTCCCTTATTTC-3` |
| *18S* | 5'-GGATGTAAAGGATGGAAAATACA-3' | 5'-TCCAGGTCTTCACGGAGCTTGTT-3' |
| *ACTB* | 5'-CATGTACGTTGCTATCCAGGC-3' | 5'-CTCCTTAATGTCACGCACGAT-3' |
| *B2M* | 5'-GAGGCTATCCAGCGTACTCCA-3' | 5'-CGGCAGGCATACTCATCTTTT-3' |
| *GAPDH* | 5'-GACAGTCAGCCGCATCTTCT-3' | 5'-TTAAAAGCAGCCCTGGTGAC-3' |
| *HPRT* | 5'-CAGTCCCAGCGTCGTGATTA-3' | 5'-TGGCCTCCCATCTCCTTCAT-3' |

**Table S3:** Pathway enrichment analysis for CA and ECM DEGs and their neighboring genes.

| GO | Category | Description | Count | % | Log10(P) | Log10(q) |
| --- | --- | --- | --- | --- | --- | --- |
| R-HSA-1474244 | Reactome Gene Sets | Extracellular matrix organization | 41 | 82.00 | -73.92 | -69.53 |
| M18 | Canonical Pathways | PID INTEGRIN1 PATHWAY | 23 | 46.00 | -49.06 | -45.15 |
| R-HSA-1474228 | Reactome Gene Sets | Degradation of the extracellular matrix | 21 | 42.00 | -35.91 | -32.48 |
| M5930 | Hallmark Gene Sets | HALLMARK EPITHELIAL MESENCHYMAL TRANSITION | 22 | 44.00 | -34.57 | -31.22 |
| GO:0001704 | GO Biological Processes | formation of primary germ layer | 16 | 32.00 | -26.60 | -23.84 |
| WP5087 | WikiPathways | Malignant pleural mesothelioma | 19 | 38.00 | -21.63 | -19.11 |
| R-HSA-202733 | Reactome Gene Sets | Cell surface interactions at the vascular wall | 14 | 28.00 | -21.19 | -18.70 |
| hsa04514 | KEGG Pathway | Cell adhesion molecules | 13 | 26.00 | -18.74 | -16.33 |
| R-HSA-3000170 | Reactome Gene Sets | Syndecan interactions | 9 | 18.00 | -18.70 | -16.30 |
| WP5055 | WikiPathways | Burn wound healing | 12 | 24.00 | -18.50 | -16.11 |
| GO:0050840 | GO Molecular Functions | extracellular matrix binding | 10 | 20.00 | -17.70 | -15.35 |
| GO:0005518 | GO Molecular Functions | collagen binding | 10 | 20.00 | -16.79 | -14.49 |
| M5915 | Hallmark Gene Sets | HALLMARK APICAL JUNCTION | 13 | 25.00 | -16.79 | -14.49 |
| R-HSA-445144 | Reactome Gene Sets | Signal transduction by L1 | 7 | 14.00 | -14.60 | -12.36 |
| GO:0005539 | GO Molecular Functions | glycosaminoglycan binding | 10 | 20.00 | -11.26 | -9.17 |
| WP3617 | WikiPathways | Photodynamic therapy-induced NF-kB survival signaling | 6 | 12.00 | -10.63 | -8.56 |
| GO:0001501 | GO Biological Processes | skeletal system development | 12 | 24.00 | -10.54 | -8.48 |
| R-HSA-76002 | Reactome Gene Sets | Platelet activation, signaling and aggregation | 9 | 18.00 | -9.34 | -7.35 |
| M22068 | BioCarta Gene Sets | BIOCARTA RECK PATHWAY | 4 | 8.00 | -9.08 | -7.12 |
| M5909 | Hallmark Gene Sets | HALLMARK MYOGENESIS | 8 | 16.00 | -8.87 | -6.91 |
| Top 20 clusters with their representative enriched terms (one per cluster). "Count" is the number of genes in the user-provided lists with membership in the given ontology term. "%" is the percentage of all of the user-provided genes that are found in the given ontology term (only input genes with at least one ontology term annotation are included in the calculation). "Log10(P)" is the *P*-value in log base 10. "Log10(q)" is the multi-test adjusted *P*-value in log base 10. | | | | | | |

**Table S4:** Pathway enrichment analysis for BCSC-related DEGs and their neighboring genes.

| GO | Category | Description | Count | % | Log10(P) | Log10(q) |
| --- | --- | --- | --- | --- | --- | --- |
| R-HSA-452723 | Reactome Gene Sets | Transcriptional regulation of pluripotent stem cells | 6 | 26.09 | -13.89 | -9.50 |
| GO:0048729 | GO Biological Processes | tissue morphogenesis | 9 | 30.00 | -8.63 | -5.67 |
| GO:0022407 | GO Biological Processes | regulation of cell-cell adhesion | 8 | 26.67 | -7.80 | -4.97 |
| GO:0008009 | GO Molecular Functions | chemokine activity | 4 | 17.39 | -7.28 | -4.52 |
| GO:0007420 | GO Biological Processes | brain development | 7 | 30.43 | -6.03 | -3.51 |
| R-HSA-6785807 | Reactome Gene Sets | Interleukin-4 and Interleukin-13 signaling | 4 | 17.39 | -5.89 | -3.38 |
| hsa05205 | KEGG Pathway | Proteoglycans in cancer | 5 | 16.67 | -5.77 | -3.30 |
| WP4659 | WikiPathways | Gastrin signaling pathway | 4 | 13.33 | -5.31 | -2.91 |
| WP4823 | WikiPathways | Genes controlling nephrogenesis | 3 | 13.04 | -5.30 | -2.90 |
| WP2023 | WikiPathways | Cell differentiation - expanded index | 3 | 13.04 | -5.13 | -2.78 |
| GO:0001666 | GO Biological Processes | response to hypoxia | 5 | 16.67 | -5.13 | -2.78 |
| M2771 | Oncogenic Signatures | ESC V6.5 UP LATE.V1 DN | 4 | 17.39 | -5.01 | -2.70 |
| M5906 | Hallmark Gene Sets | HALLMARK ESTROGEN RESPONSE EARLY | 4 | 17.39 | -4.82 | -2.54 |
| WP5094 | WikiPathways | Orexin receptor pathway | 4 | 13.33 | -4.78 | -2.52 |
| GO:0010817 | GO Biological Processes | regulation of hormone levels | 5 | 16.67 | -3.92 | -1.86 |
| R-HSA-71387 | Reactome Gene Sets | Metabolism of carbohydrates | 4 | 13.33 | -3.70 | -1.68 |
| R-HSA-9006931 | Reactome Gene Sets | Signaling by Nuclear Receptors | 4 | 13.33 | -3.68 | -1.66 |
| hsa05166 | KEGG Pathway | Human T-cell leukemia virus 1 infection | 3 | 10.00 | -2.86 | -0.97 |
| WP2882 | WikiPathways | Nuclear receptors meta-pathway | 3 | 10.00 | -2.40 | -0.59 |
| Top 20 clusters with their representative enriched terms (one per cluster). "Count" is the number of genes in the user-provided lists with membership in the given ontology term. "%" is the percentage of all of the user-provided genes that are found in the given ontology term (only input genes with at least one ontology term annotation are included in the calculation). "Log10(P)" is the *P*-value in log base 10. "Log10(q)" is the multi-test adjusted *P*-value in log base 10. | | | | | | |

**
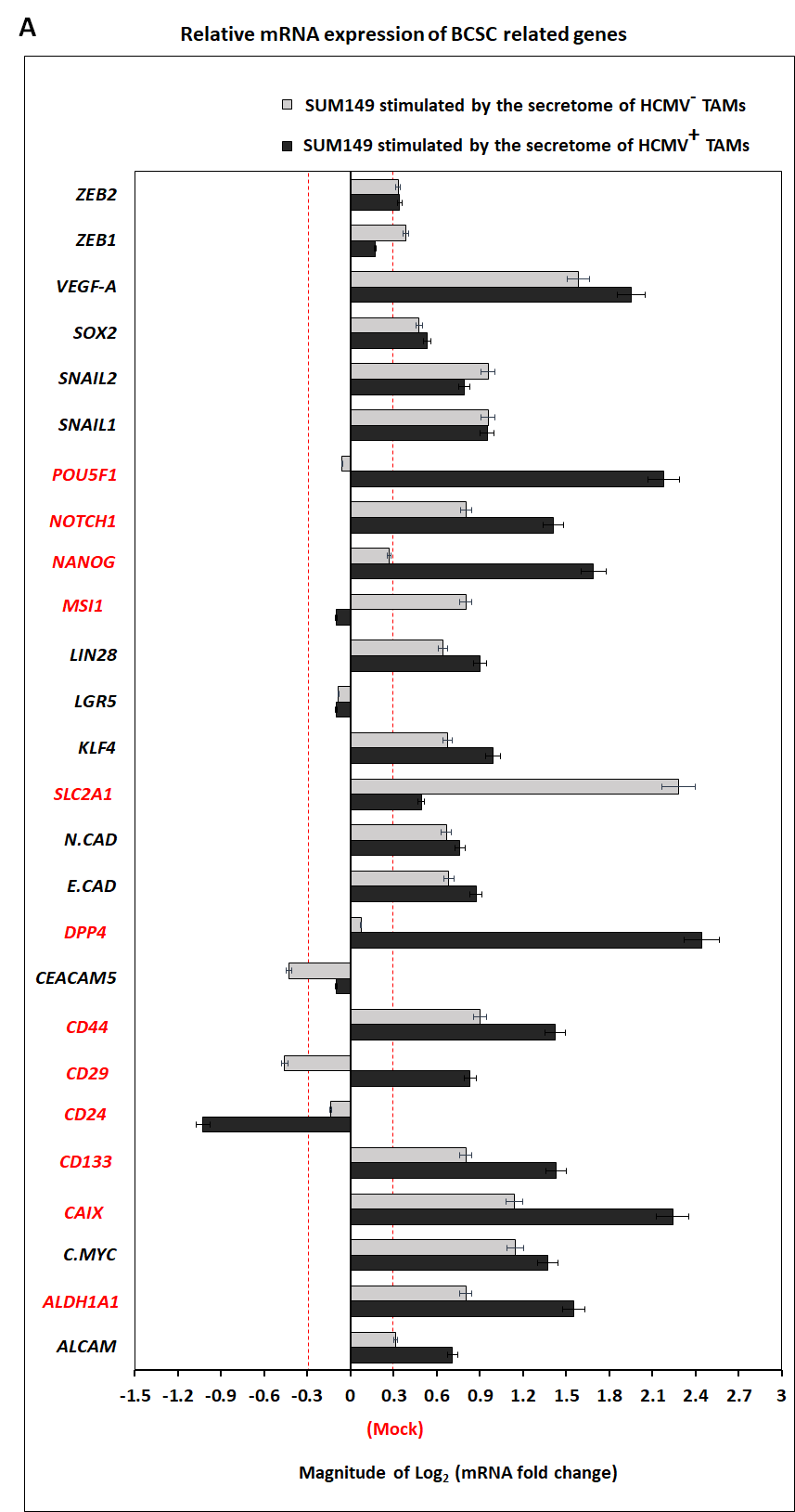
Fig. S1: Secretome of HCMV^+^ TAMs upregulate BCSC-related genes in SUM149 cells.** (A) Clustered bars represent the mRNA expression of BCSC-related genes in SUM149 cells stimulated by the secretome of HCMV^-^ and HCMV^+^ TAMs compared to mock cells.
